# Yogurt fortification by microencapsulation of beetroot extract (*Beta vulgaris* L.) using maltodextrin, gum arabic, and whey protein isolate

**DOI:** 10.1002/fsn3.2804

**Published:** 2022-05-11

**Authors:** Shima Yousefi, Mohammad Kavyanirad, Mehrnaz Aminifar, Weria Weisany, Amin Mousavi Khaneghah

**Affiliations:** ^1^ 125643 Department of Agriculture and Food Science Islamic Azad University Science and Research Branch Tehran Iran; ^2^ Standard Research Institute – SRI Karaj Iran; ^3^ Department of Food Science and Nutrition Faculty of Food Engineering University of Campinas (UNICAMP) Campinas, São Paulo Brazil

**Keywords:** beetroot juice, microencapsulation, spray‐drying, stirred yogurt

## Abstract

The effect of three different coating materials, including maltodextrin (MD, 9.95–20.05%), gum arabic (GA, 4.98–10.02%), and whey protein isolate (WPI, 4.95–15.05%), was optimized in order to produce high‐quality beetroot extract powder (BEP) using response surface modeling (RSM). Beetroot extract (BE) was encapsulated using MD, GA, and WPI by implementing a spray‐drying method. The highest total phenolic content (TPC) was obtained at 15% MD, 7.5% GA, and 10% WPI. The same results were achieved for antioxidant activity. Increasing the MD and GA contents resulted in reducing the moisture adsorption of microencapsulated BEP.

## Graphical abstract

Beetroot extract (BE) was encapsulated using maltodextrin (MD), gum arabic (GA), and whey protein isolate (WPI) by implementing a spray‐drying method. The highest total phenolic content (TPC) was obtained at 15% MD, 7.5% GA, and 10% WPI. The same results were achieved for antioxidant activity. Increasing the MD and GA contents resulted in a reduction of moisture adsorption of microencapsulated BE powder.

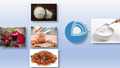

## 1 INTRODUCTION

Beetroot (*Beta vulgaris* L.) is one of these plant‐rich types in anthocyanins and polyphenolic compounds (Gengatharan et al., 2015). Red beetroot is mainly used as a natural dye in food recipes. An appropriate ratio of ethanol and water is usually used to extract anthocyanins and other efficient ingredients. The dominant part of the extract is composed of anthocyanins (known as betalains) which comprise two groups: betaxanthins (e.g., vulgaxhantin I and II) and betacyanins (e.g., betanin and isobetanin), which are responsible for the yellow and violet‐red hues, respectively (Cardoso‐Ugarte et al., 2014; Gengatharan et al., 2015). However, natural additives are sensitive and may miss their initial effects. Betalains are stable over a pH range of 3–7, which can be incorporated in a wide variety of foods. In contrast, they possess low thermal stability and are reactive with oxidizing agents (oxygen, light). Therefore, increasing its chemical stability in the food matrix is necessary. During the spray‐drying process, the atomized droplets may adhere to the chamber wall, and then the active agents would be thermally damaged. This can be attributed to low‐molecular‐weight sugars and organic acids, with low glass transition temperature (*T_g_*). Also, hot air flows in the drying chamber and atomized droplets expose to high temperatures (>130°C, the inlet temperature). Although instant moisture evaporation occurs, applying high temperatures may negatively impact the bioactivity of food ingredients or nutraceutical compounds (Rezvankhah et al., 2020). Hence, the process conditions, including feed pump rate, air pressure, aspiration ratio, and inlet and outlet temperatures, should be optimized (Rezvankhah et al., 2020). Besides, emulsions or fluids should be enriched with biopolymers before atomizing through the chamber to increase the *T_g_* and give thermodynamically stable emulsions (Rezvankhah et al., 2020). Biopolymers, including carbohydrates and proteins, have been extensively used to encapsulate food ingredients (Mansour et al., 2020). Maltodextrin (MD), modified starches, gum arabic (GA), and chitosan have the most consumption among the carbohydrates, while whey protein (isolate and concentrate) and sodium caseinate, and generally milk proteins, have been widely used to embed the bioactive or nutraceutical components (Arabpoor et al., 2021; Jia et al., 2016; Mahmmodi et al., 2021; Zhu, 2017). The combination of biopolymers can efficiently encapsulate nutraceuticals, and produced powders would have higher storage stability (Mansour et al., 2020).

Whey protein isolate (WPI), a primary by‐product of the dairy industry, has been extensively used individually or in combination with other polysaccharides as coating materials for encapsulation due to the good emulsifying characteristics and ability to form a strong gel matrix.

MD, a starch derivative product, possesses a wide range of solubility, good morphology, low viscosity at high concentrations, and great controlled release ability (Tolun et al., 2016). Although MD has been widely used in food industries, it does not provide an excellent emulsifying ability and, therefore, could be used with a practical carrier agent to improve the emulsifying properties (Tolun et al., 2016).

Over the last few years, the most common emulsifying agent used in the food industry is GA. GA, a mixture of polysaccharides and glycoproteins (GPs), was utilized as a microencapsulating agent to protect bioactive compounds due to its high‐amphiphilicity and low viscosity and its good emulsifying characteristics (Daoub et al., 2018).

The main aim of the present study was the optimization of beetroot extract (BE) encapsulation using the RSM method. RSM has been successfully used for developing, improving, and optimizing processes. MD, GA, and WPI were used as coating materials, and the influence of various formulations on the attributes of the BE microcapsules was evaluated. Finally, the stirred yogurt was formulated with obtained BEP at different concentrations and determined Beetroot flavored yogurt's physicochemical properties.

## 2 MATERIALS AND METHODS

### 2.1 Materials

Beetroot was purchased from the local market (Tehran, Iran). Hydrocolloids, including MD with a high dextrose equivalent (DE; 18–20), GA, WPI, and also other chemical solvents and compounds, all were purchased from Merck Co. (Germany).

### 2.2 Extraction of anthocyanins

According to the procedure reported in the literature, the conventional maceration method was applied to extract anthocyanins (Tiwari et al., 2010).

### 2.3 Encapsulation of anthocyanins

After the achievement of anthocyanins extract, the feed solution for spray‐drying was prepared according to Table 1. The obtained extract was well‐mixed with biopolymer solutions using Ultra‐Turrax homogenizer (T 25 digital ULTRA‐TURRAX^®^, Germany). Based on the pretreatments, the optimum spray‐drying condition was obtained as follows: inlet temperature of 136°C, the outlet temperature of 90–110°C, the feed rate of 8.8 ml/min, and airflow of 3.6 m^3^/h. Eventually, the obtained microencapsulated powders were stored in dark glass containers at ambient temperature.

**TABLE 1:** Experimental design for preparation of beetroot extract powder using the RSM‐CCRD used for the spray‐drying treatments

Run	WPI (%)	MD (%)	GA (%)
1	7	12	6
2	7	12	9
3	7	18	6
4	7	18	9
5	13	12	6
6	13	12	9
7	13	18	6
8	13	18	9
9	10	15	4.98
10	10	15	10.02
11	10	9.95	7.5
12	10	20.05	7.5
13	4.95	15	7.5
14	15.05	15	7.5
15	10	15	7.5
16	10	15	7.5
17	10	15	7.5
18	10	15	7.5
19	10	15	7.5
20	10	15	7.5

Abbreviations: GA, gum arabic; MD, maltodextrin; and WPI, Whey protein isolate.

### 2.4 Powder yield

Powder yield was determined as the ratio of the mass of total solids in the produced powder to the mass of total solids in the feed solution (Santana et al., 2016).

### 2.5 Determination of encapsulation efficiency (EE%)

Encapsulation efficiency was determined according to the method announced by Otálora et al. (2015). Total betalains content (TBC) and surface betalains content (SBC) were calculated for this test. TBC was calculated as betanin (betanidin 5‐O‐β‐d‐glucoside) according to the following equation:

$$\mathrm{TBC}\;\left(\frac{\mathrm{mg}}{L}\right)=\Delta A\varepsilon \times 1\times M\times 103\times D$$

where ∆*A*, (A520 pH 1‐A700 pH 1) – (A520 pH 4.5‐A700 pH 4.5); ε (molar extinction coefficient = 65,000 L/mol cm for betanin; 1, path length in cm; *M* (molecular weight) = 550.1 g/mol for betanin; *D*, dilution factor; 103, conversion from gram to milligram.

### 2.6 Characterization of microencapsulated powders

#### 2.6.1 Moisture content

The moisture content of microcapsules was determined gravimetrically by drying 1 g of samples at 65°C, in triplicate, until constant weight (AOAC, 2000).

### 2.7 Bulk density

Two grams of samples was weighed and poured in a 10 ml graduated cylinder and then kept for 1 min on a vibration vortex. The weight of powders to the volume occupied in the cylinder was bulk density (g/ml) (Karaaslan & Dalgıç, 2014).

### 2.8 True density

The True density was calculated using a pycnometer and ethanol 44% as an immiscible solvent. Also, the porosity of powders was obtained by the following equation (Akhavan Mahdavi et al., 2016):

$$\varepsilon =1‐\frac{\mathrm{pb}}{\mathrm{pt}}$$

where pt and pb were true density and bulk density, respectively.

### 2.9 Solubility

Two grams of powders was added to 100 ml distilled water in a beaker and mixed using a homogenizer at 25,200 *g* for 5 min. The obtained mixture was centrifuged at 3000 ***g***, and the separated supernatant (25 ml) was poured into a preweighed plate. Then, the plate was transferred to an oven set at 105°C for 4 h for drying. The solubility was computed based on the difference between the obtained weight (Fazaeli et al., 2016).

### 2.10 Hygroscopicity

One gram of samples was weighed in a container with a certain weight and then placed in a desiccator containing a saturated solution of NaCl at ambient temperature. After a week (moisture equilibrium), the containers were weighed, and the moisture adsorption was determined by the following equation:

$$\mathrm{Hy}\;\left(\%\right)=\frac{\left(\left(W_{2}‐W_{1}‐W_{0}\right)\times 1000\right)+\left(W_{1}\times M\right)}{(W_{2}‐W_{0})\times 10}$$

Hy (%) was moisture adsorption (g of adsorbed moisture/100 g powder); *w*_0_ was empty container (g); w_1_ was the weight of power (g); w_2_ was the weight of powder with container after moisture equilibrium; *M* was moisture content of powder (g/kg powder).

### 2.11 Flowability

The flowability of powders was calculated according to the Hausner ratio. Ten grams of powders was weighed in a graduated cylinder. The initial volume was noted (*V*_b_), and then the cylinder was tapped to reach a constant volume (*V*_f_). Finally, the flowability was calculated by the equation below (Akhavan Mahdavi et al., 2016):

$$\mathrm{Hausner}\;\mathrm{ratio}\;\left(\mathrm{HR}\right)=\left(\frac{V_{b}}{V_{f}}\right)\times 100$$

Flowability is denoted based on the different ranges of Hausner ratio:

HR between 1 and 1.1: free‐flowing powder; HR between 1.1 and 1.25: medium flowing powder; HR between 1.25 and 1.4: difficult flowing powder; HR higher than 1.4: very difficult flowing powder.

### 2.12 Morphological properties

The morphological properties of samples were examined by a Zeiss DSM 960 digital scanning electron microscopy (SEM) (Zeiss, Oberkochen, Germany) operated under a voltage of 26 Kv (Mestry et al., 2011).

### 2.13 Antioxidant power

The antioxidant power of powder samples was determined according to the method reported by Yousefi et al. (2012) with slight modification. 2, 2‐Diphenyl‐1‐picrylhydrazyl (DPPH) was used as free reactive radicals to evaluate the antioxidant activity. The 0.5 g of samples was dissolved in 25 ml of methanol, and time was given to extract anthocyanins. Then, the obtained suspensions were centrifuged, and the supernatants were separated. In the next step, 1 ml of supernatant was mixed with 3 ml of DPPH solution (0.025 ppm), and the reaction was conducted for 40 min in a dark place. Finally, the change in the absorption of solutions at 515 nm was recorded by using spectrophotometer UV‐vis. Radical scavenging activity power was calculated by the equation below:

$$\mathrm{DPPH}\;\left(\%\right)=\frac{A_{s}}{A_{b}}\times 100$$

where *A_s_* and *A_b_* were the absorbances of sample and blank solutions, respectively.

### 2.14 Total phenol content

Total phenol content (TPC) of powder samples was measured by the Folin–Ciocalteu method based on that announced by Bansal et al. with brief modification. An amount of 0.5 g of powder was dissolved in 25 ml of methanol, and after a while, polyphenols were extracted; the solutions were centrifuged for 10 min. About 0.5 ml of supernatant was mixed with 2.5 ml of Folin–Ciocalteu reagent 0.2 N, and the reaction was conducted for 5 min. Then, 2 ml of sodium carbonate solution (75 g/L) was added to the reaction mixture, and the volume was reached 25 ml by the addition of distilled water. The obtained solutions were placed at ambient temperature for 2 h, and the absorbance of samples was read using spectrophotometer UV‐vis at 760 nm. Gallic acid solutions (0–100 mg/L) were used to plot the standard diagram. TPC was expressed as mg gallic acid equilibrium/g of powder (GAE/g powder). All determinations were implemented in three replications.

### 2.15 Experimental design

Design expert version 11 was used to optimize the encapsulation process of anthocyanins obtained from red beetroot. MD, GA, and WPI were considered as independent variables (Table 1). In the next step, based on the preliminary study, different levels of microencapsulated BEP (0, 0.5, and 1%) were used to fortify stirred yogurt with different amounts of fat (1.5, 2, and 3.2%). Further, the physicochemical properties of the developed product were also evaluated and compared with control during storage for one week (1, 3, 5, and 7 days). All experiments were carried out in triplicate.

## 3 RESULTS AND DISCUSSION

### 3.1 Powder yield (%)

**FIGURE 1:**
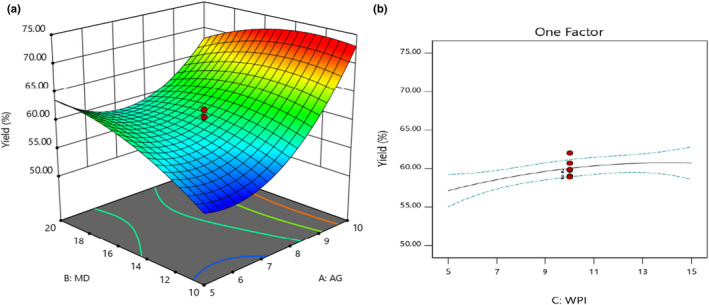
Effects of MD, GA, and WPI on the powder yield (%)

According to **Figure** **1**, GA had a higher effect than MD in increasing powder yield. Based on statistical analysis (ANOVA), linear and quadratic effects of GA were more impressive than the exact effects of MD (data not shown). Also, it was observed that the simultaneous use of MD and GA had a higher effect than individually utilizing MD. On this basis, their interaction was obtained significantly (*p* < .05). The highest amount (72%) was achieved when 10% GA and 15% MD were used. Similar results were discovered by Akhavan Mahdavi et al. (2016) and Burin et al. (2011), who studied microencapsulation of natural anthocyanins by MD, GA, gelatin, and MD, MD/γ‐cyclodextrin, and MD/GA, respectively. According to **Figure** **1**, by an increase in WPI level from 5% to 15%, the powder yield was slightly increased from 57% to 60%. Statistical analysis presented only linear terms of WPI significance. The interaction effects between WPI and GA‐MD were not significant (*p* > .05). Generally, fruit juices are intrinsically sticky and reduce the spray‐drying powder yield. It is due to the low *T_g_* point of natural juices. To master this drawback, polysaccharides, gums, and proteins can increase the *T_g_* point of provided emulsions and reduce adherence. MD forms a nonsticky film around the particles of the feed solution and reduces the adhesion. Zareifard et al. (2012) announced that MD could reduce the sticky property of lime juice during the spray‐drying process.

### 3.2 Chemical properties of microcapsules

#### 3.2.1 TPC

**FIGURE 2:**
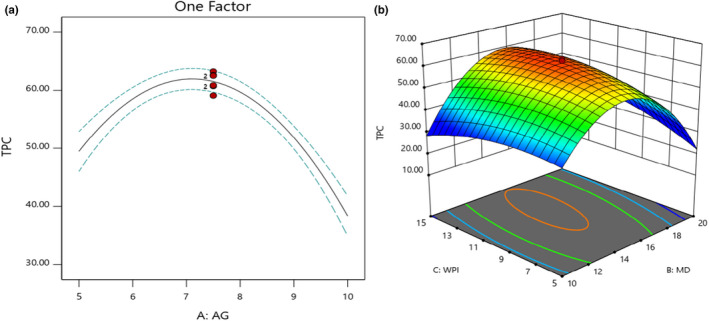
Effects of MD, GA, and WPI on the TPC of obtained microencapsulated powders

According to the ANOVA analysis (not shown), the model showed high significans and good fit with the TPC values (*p* < .05). TPC results indicated that microcapsules depending on their compositions would have different values. The TPC increased with the increasing GA concentration up to 7.5% and was followed by a decrease afterwards (Figure 2a). MD when was increased from 10% to 15%, TPC was increased, and the highest amount of TPC was achieved when 15% MD and 10% WPI was used. Based on statistical results, the linear effects of WPI and GA were significant (*p* < .05; Figure 2b). Also, the interaction terms of MD‐WPI were obtained significantly (*p* < .05).

All quadratic terms were significant despite GA and MD having such an impressive influence on the TPC. Among the various carriers used to preserve the bioactive compounds, MD had a higher *T_g_* point, which causes less adhesiveness of produced powders to the drying chamber wall (Goula & Adamopoulos, 2012). However, it suffers from low surface activity. GA is mainly composed of guluronic acid and has partial protein that has been covalently linked to carbohydrate chains and causes a good film in the emulsions (Rezvankhah et al., 2020). It is mainly used with MD or modified starch for a synergistic effect (Akhavan Mahdavi et al., 2016; Santana et al., 2016). As Santana et al. (2016) reported, the use of GA and modified starch together with either WPC or WPI provide better microencapsulation efficiency. Indeed, GA is a surface‐active agent and even can be more effective than MD and WPI. GA can lonely give rise to high encapsulation efficiency. MD due to having low emulsifying activity and low ability in film formation would give rise to low encapsulation efficiency if it were used lonely. Hence, it is suggested that MD should be used along with a surfactant, either with WPI or GA (Assadpour & Jafari, 2019; Rezvankhah et al., 2020).

### 3.3 DPPH radical scavenging activity

**FIGURE 3:**
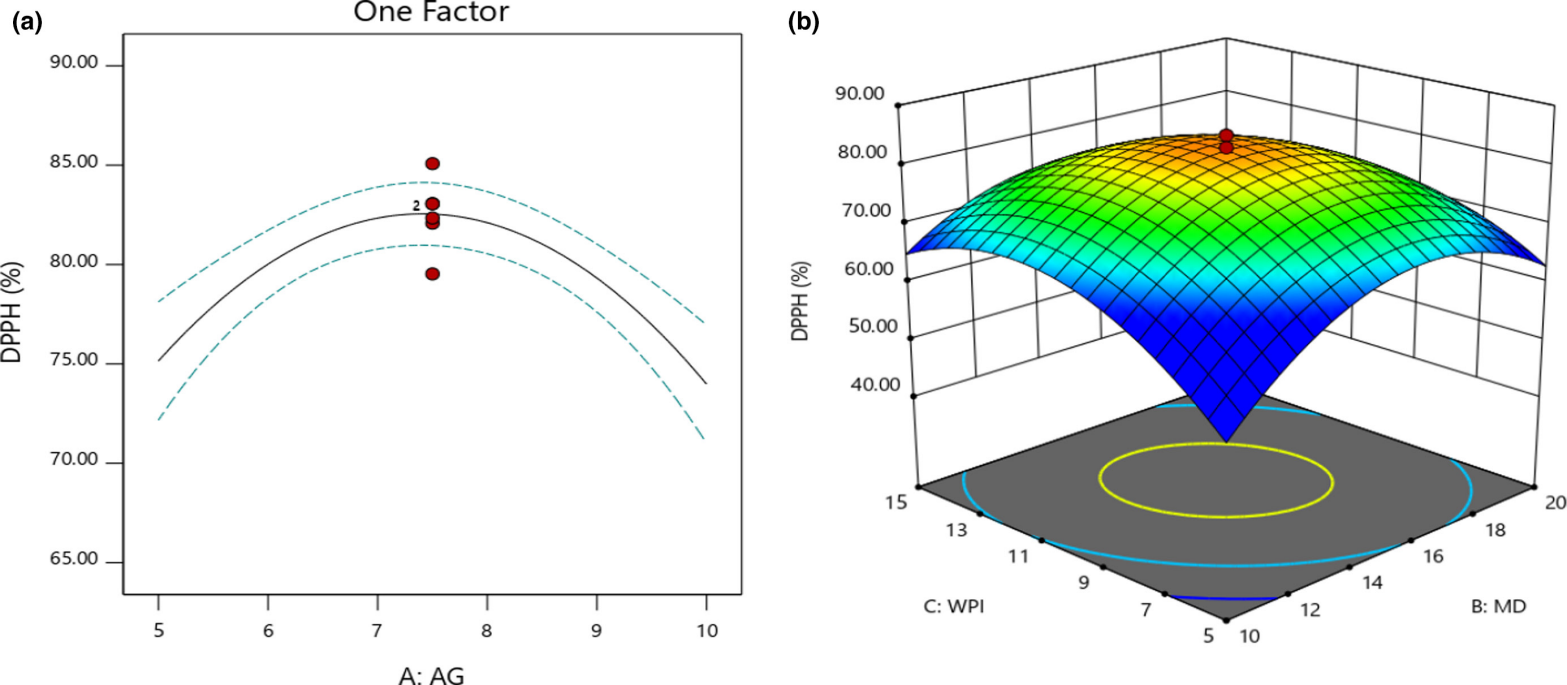
Effects of MD, GA, and WPI on the DPPH radical scavenging activity (%) of microencapsulated powders

According to Figure 3a, when the concentration of GA was increased from 5 to 7.5%, the antioxidant activity of powder was increased (75–85%). Also, MD and WPI enhancement from 10 to 15 and 5 to 10%, respectively, increased the antioxidant activity of microencapsulated powders (Figure 3b). A moderate amount of biopolymers would result in higher antioxidant activity. The excess of biopolymers leads to depleted flocculation ( Rezvankhah et al., 2020). Indeed, biopolymers react with each other and do not involve emulsion formation. Due to having a higher *T_g_* point, MD reduces the adhesion of dried powder to the drying chamber, and subsequently, the deterioration effect on the bioactive compounds would be decreased. Furthermore, whey protein also has been reported to have antioxidant activity, which can be related to its sulfhydryl (‐SH) groups that can reduce free radicals (Premi & Sharma, 2017).

### 3.4 Physical properties of microencapsulated powders

#### 3.4.1 Moisture content

The moisture content of powders was determined based on the difference of initial mass and secondary mass (constant weight) obtained after oven drying at 104°C for 2 h.

### 3.5 Moisture adsorption

**FIGURE 4:**
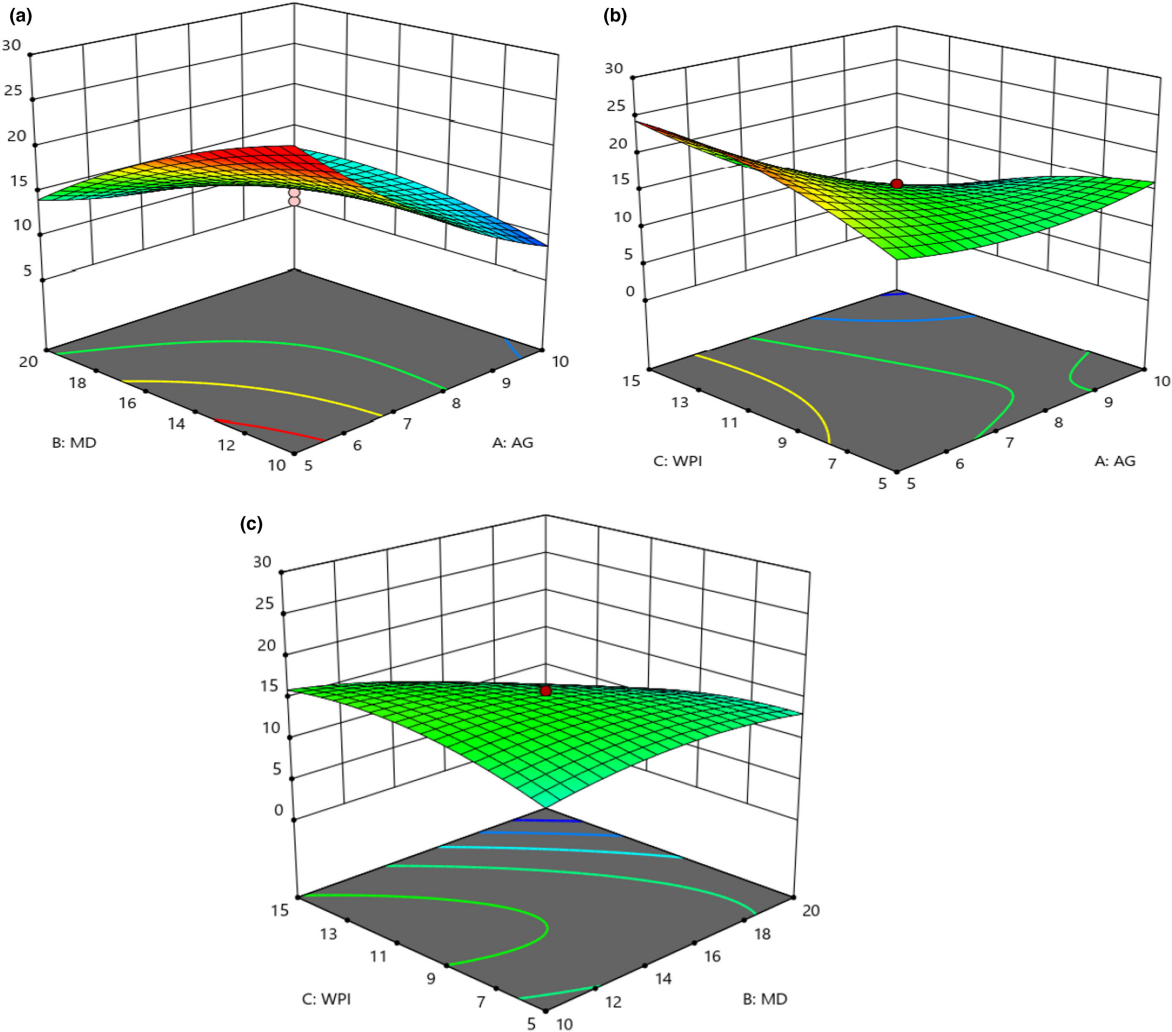
Effects of MD, GA, and WPI on the moisture adsorption

According to Figure 4a, an increase in MD and GA concentrations reduced the moisture adsorption where the effect of GA was higher than MD. On the other hand, an increase in WPI concentration increased the moisture adsorption of microencapsulated powders, while the combination with GA reduced respective values (Figure 4b). Based on the interaction effect of MD and WPI, an increase in concentrations significantly reduced the moisture adsorption of microencapsulated powders (*p* < .05; Figure 4c). Generally, moisture adsorption is the ability of powders to absorb moisture in a medium with high relative humidity. Powders with low moisture absorption, low moisture content, low degree of caking, and high solubility are considered desirable and longer shelf life. The moisture adsorption values were 8–24%. By increasing of *T_g_* point, moisture adsorption is reduced. Thus, the increase of carriers' concentration may increase the *T_g_* point, which results in a reduction of moisture adsorption.

### 3.6 Flowability of microencapsulated powders

**FIGURE 5:**
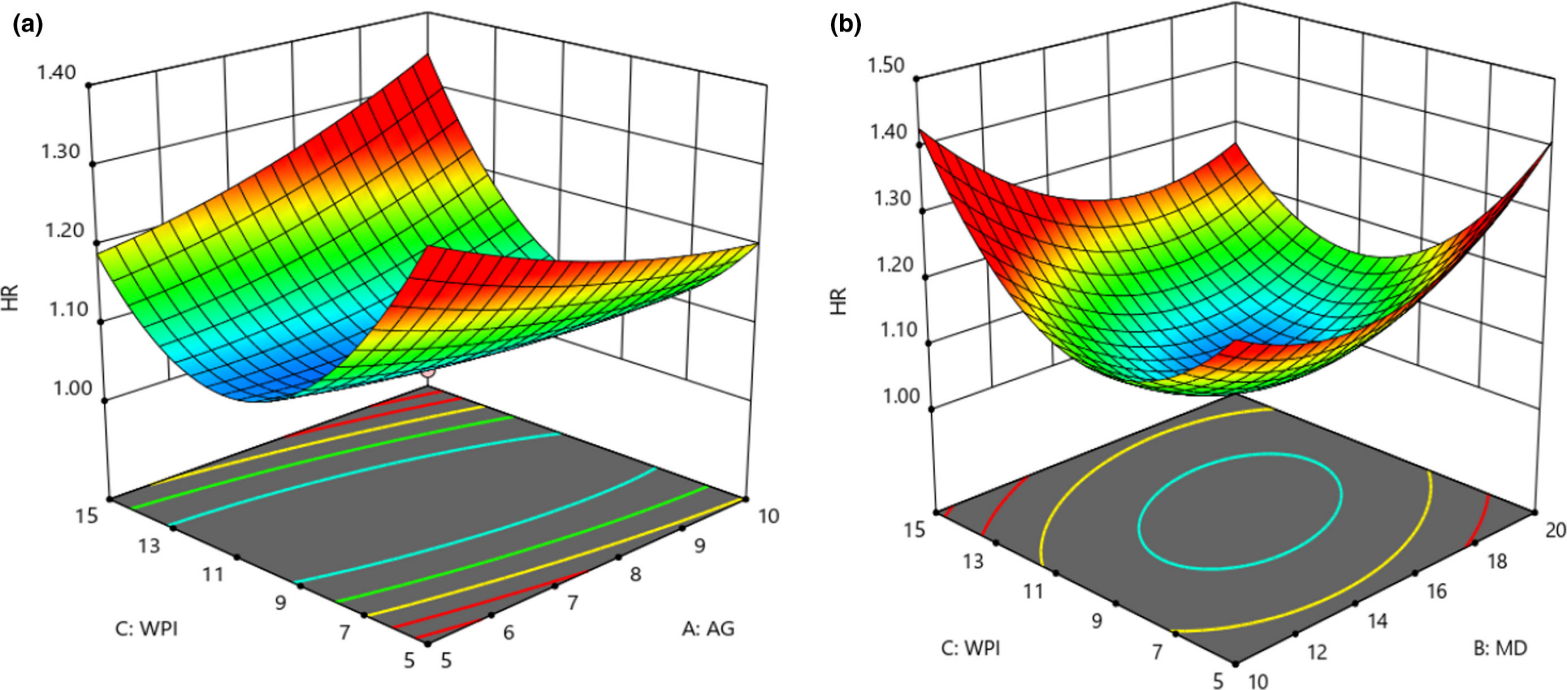
Effects of MD, GA, and WPI on the flowability of microencapsulated powders

Based on the obtained results, the interaction effect of GA and WPI showed that the highest flowability value was attributed to powder with 10% GA and 15% WPI (Figure 5a). An increase in WPI concentration from 5 to 10% has reduced the flowability, while an increase from 10 to 15% increased the flowability value. Also, the interaction effect of MD and WPI indicated that the combined effect significantly influenced the flowability of powders, and respective values were decreased, which was attributed to the high moisture content of powders (*p* < .05; Figure 5b). Moreover, the quadratic effects of both MD and WPI concentrations were significant, and the flowability of powders was increased (*p* < .05; Premi & Sharma, 2017).

### 3.7 Bulk density

**FIGURE 6:**
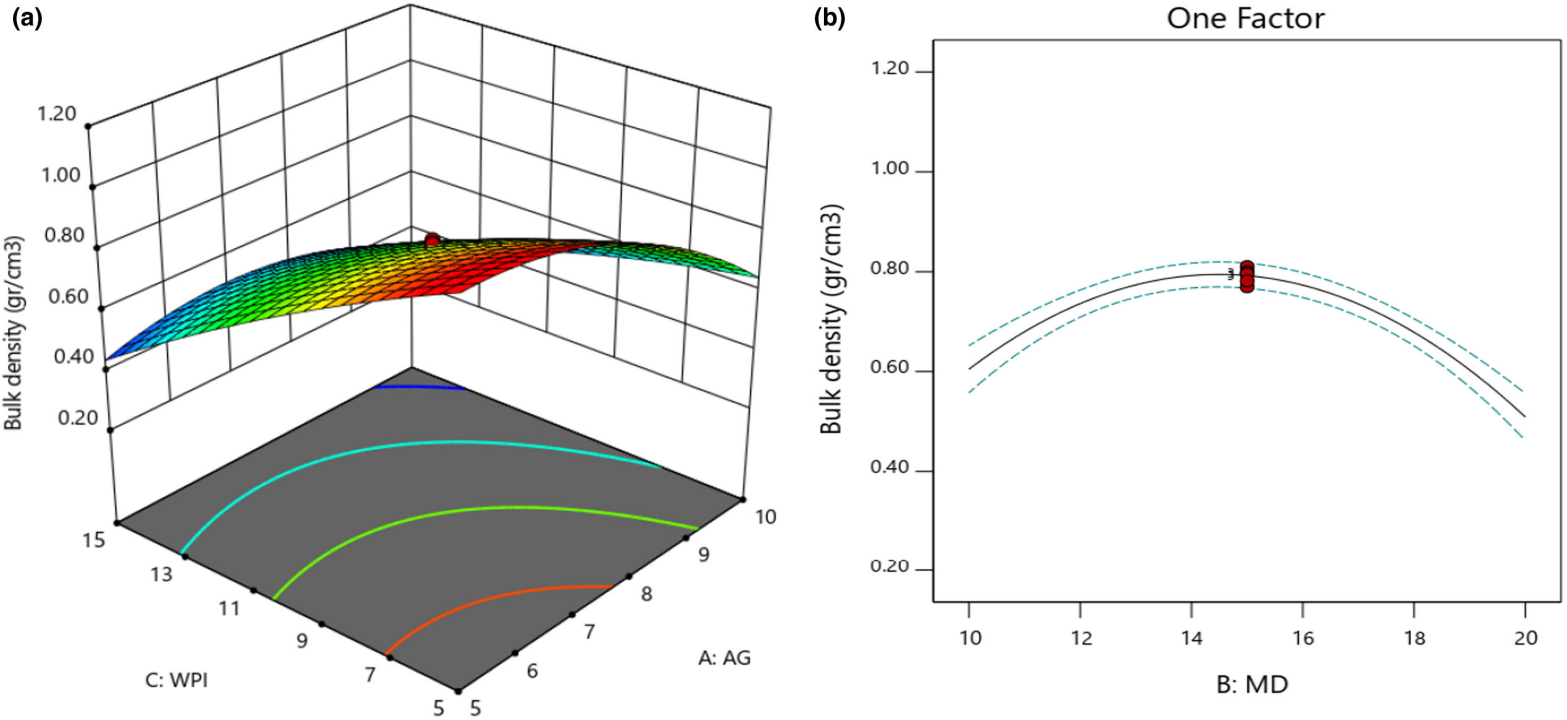
Effects of MD, GA, and WPI on the bulk density of microencapsulated powders

Bulk density is related to the molecular weight of wall materials. The heavier the material, the more quickly it accommodates into the spaces between the particles, occupying less space and resulting in higher bulk density values (Akhavan Mahdavi et al., 2016; Premi & Sharma, 2017). On this basis, the obtained bulk density results for microencapsulated powders showed that an increase in the concentration of GA and WPI led to a reduction of bulk density (Figure 6a). WPI caused a reduction of bulk density of powders more than GA. The lowest bulk density was obtained when 10% of GA and 15% of WPI were utilized. According to Figure 6b, an increase in MD concentration until 15% caused enhancement of bulk density while an increase from 15 to 20% reduced the bulk density. The lowest bulk density (0.51 g/cm^3^) was obtained when 20% of MD was applied. Bulk density is one of the important parameters that is determined for powders. It is substantial regarding transportation, storage, and packaging. Bulk density depends on the particle size, particle size distribution, moisture content, chemical composition, and the entrapped air inside the particles. These depend on feeding properties, airflow, inlet and outlet temperature, drying time, processing stage, and transportation. Smooth and uniform powders have a higher bulk density.

The viscosity of feed has a crucial role in the density of powders. Whatever the viscosity of feed is increased, larger particles would be produced. The larger the particle size, the more space between the particles, thereby reducing bulk density. Also, the lower the bulk density is, the higher the solubility is (Fazaeli et al., 2012). The molecular weight of carriers is effective in the bulk density of powder. According to Goula and Adamopoulos (2005), the bulk density of powders with high GA content was higher than those with a high amount of MD. Regarding the smaller molecular size of GA compared to MD, smaller particles are produced (low volume of powder) when a high amount of GA is applied, and therefore, density increases.

### 3.8 Absolute density

**FIGURE 7:**
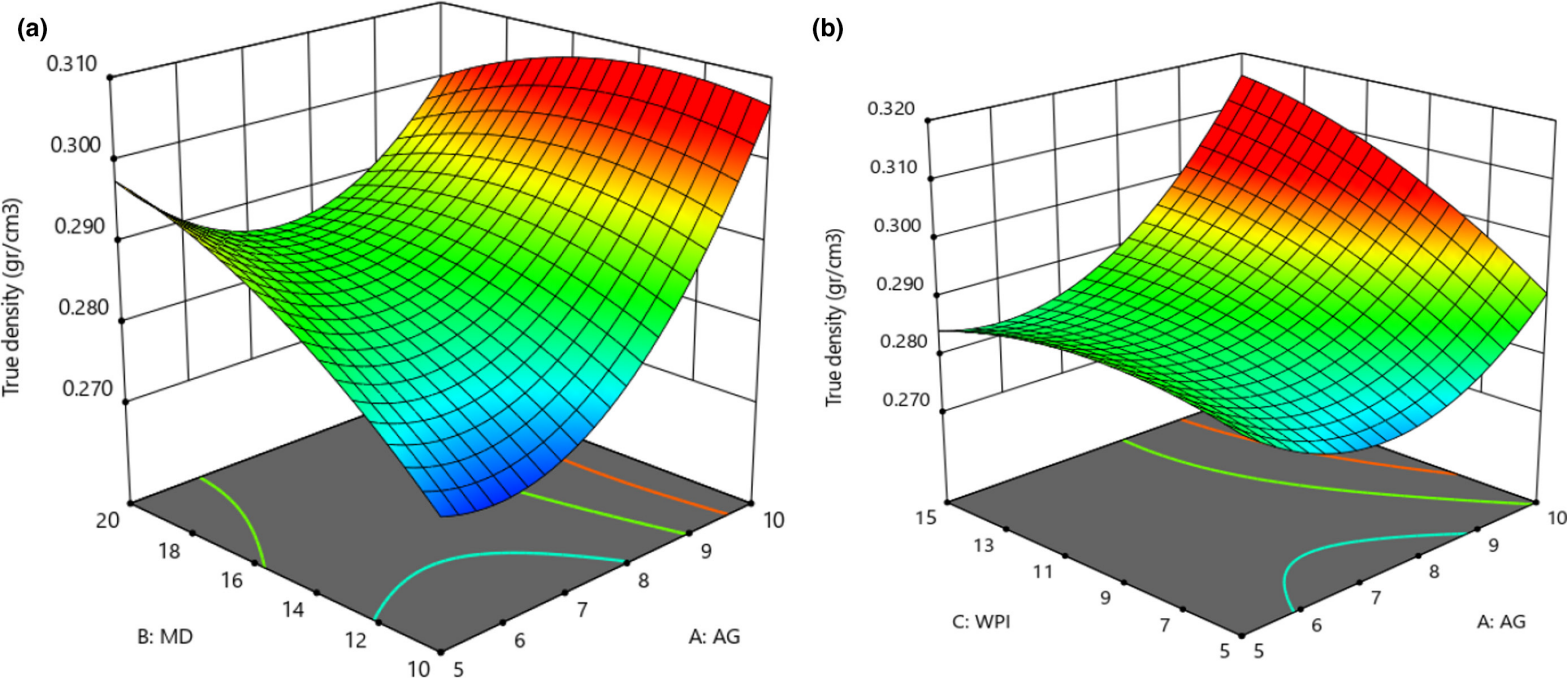
Effects of MD, GA, and WPI on the absolute density of microencapsulated powders

Figure 7a illustrates the significant interaction effect of GA and MD on the absolute density of red beetroot microencapsulated extract (*p* < .05). It was revealed that an increase in GA and MD amounts enhanced the absolute density, and the effect of GA was higher than MD, which was in agreement with ANOVA analysis (not shown; *p* < .05). The interaction effect of GA and WPI was also significant, and an increase in their concentrations enhanced the absolute density (Figure 7b). According to ANOVA analysis (not shown), the quadratic effect of GA was only obtained significant (*p* < .05), which implied that GA was so impressive on absolute density. The highest density was obtained when 10% of GA and 15% of WPI were incorporated.

### 3.9 The porosity of microencapsulated powders

**FIGURE 8:**
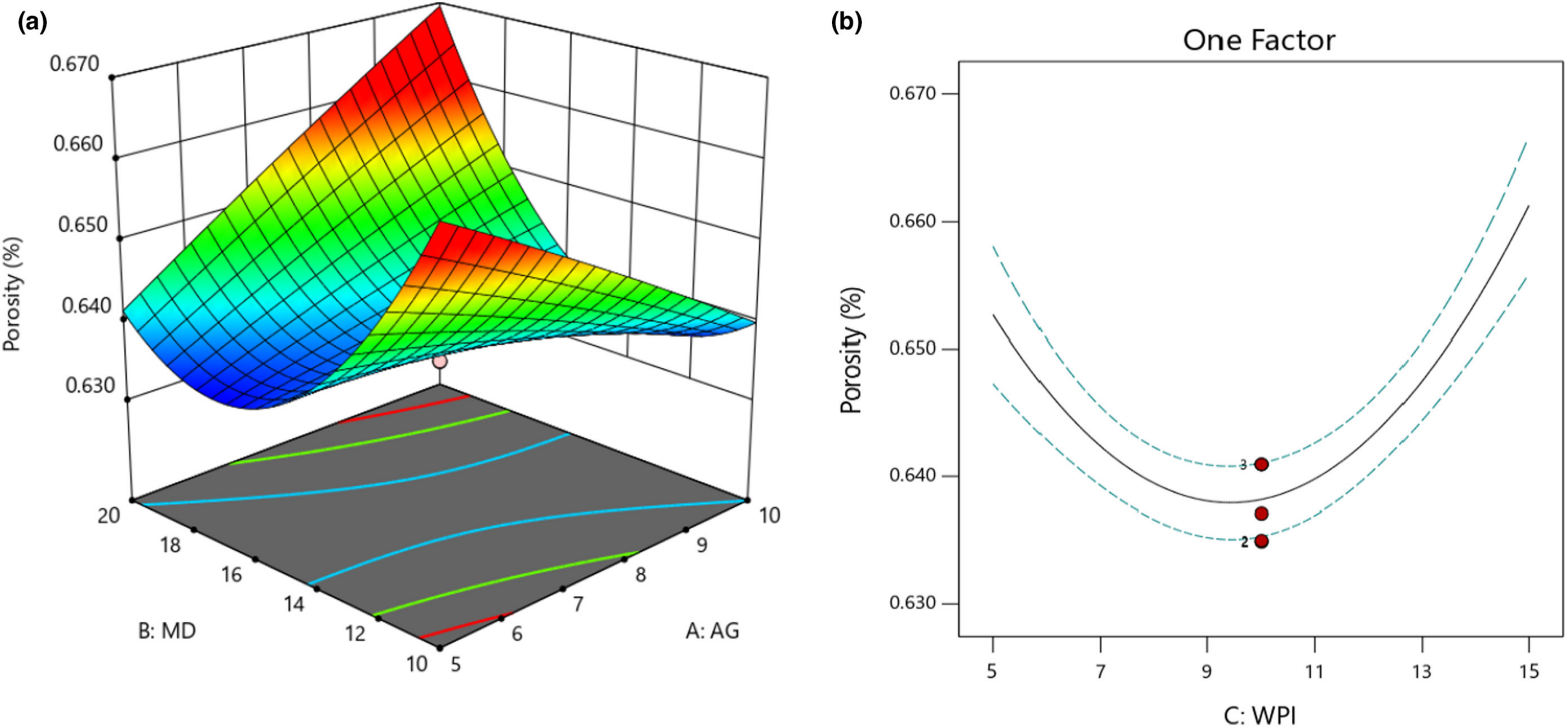
Effects of MD, GA, and WPI on the porosity of microencapsulated powders

According to results, GA and MD individually reduced the porosity of powders, while based on the interaction effect, the simultaneous utilization of MD and GA significantly increased the porosity (*p* < .05; Figure 8a). It is necessary to declare that GA's linear and quadratic effects were insignificant (*p* > .05). The highest porosity was obtained when 10% of GA and 20% of MD were used. On the other hand, WPI had a significant effect on the porosity, linearly and quadratically (*p* < .05) (Figure 8b). WPI until 10% reduced the porosity and from 10 to 15% caused increment of porosity values.

### 3.10 Optimization

Response surface methodology aims to find the most desirable point. This point depends on the aim that is following. It can be a maximum value for a particular response or a minimum value for other responses. In the present study, optimization was conducted based on maximum lightness, redness, TPC, antioxidant power, flowability, porosity, and the minimum bulk density values, yellowness, and moisture adsorption. The optimum point was obtained when 9.19% of GA, 15.61 of MD, and 14.37% of WPI were used. It had a desirability of 0.7.

### 3.11 *SEM* analysis

**FIGURE 9:**
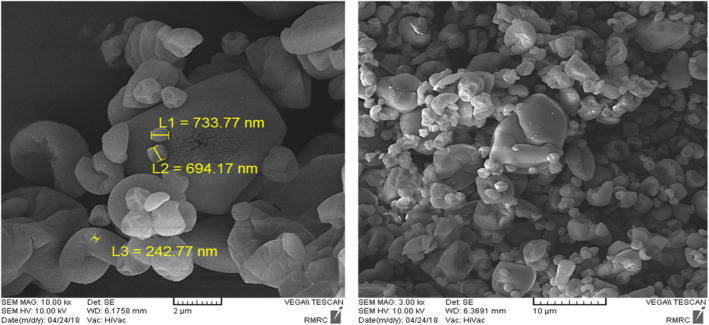
Morphological properties of microencapsulated powder

Figure 9 indicates the morphological properties of microencapsulated powders. The produced particles exhibit rough surfaces and a slight shrinkage related to the high temperature applied during the drying process. A large number of particles have been agglomerated due to physical instability, while small particles were also remain separated. Based on Figure 9, the produced microencapsulated with GA had a spherical and smooth structure with a smaller particle size (100–400 nm). In contrast, the powders produced with WPI had shrieked, rough, serrated, and agglomerated surface, which was attributed to partial denaturation of protein molecules during the initial stage of drying, where the slow formation of shell around the bioactive cores occurred (Rezvankhah et al., 2020).

### 3.12 Physiochemical characteristic of yogurt with microencapsulated BEP

#### 3.12.1 Syneresis

According to Table 2, the inclusion of microencapsulated powder (0.5 and 1%) into yogurt with different fat contents (1.5, 2, and 3.2%) significantly reduced the syneresis during the storage time (1, 3, 5, and 7; *p* < .05). On day 1, the highest syneresis amount was related to the plain sample with 2% fat content, and the lowest amount was obtained for the sample with 1% powder and 1.5% fat content. Regarding the samples without powder, when the fat content was increased from 1.5 to 2%, the syneresis was increased, while when the fat content was reached 3.2%, the syneresis was reduced. On day 3, the highest syneresis value was related to yogurt with 0.5% powder and 3.2% fat content, while the lowest syneresis value was related to sample 1% of powder and 1.5% of fat content. On day 5, the highest syneresis value was related to the plain sample with 3.2% fat content, while the lowest was related to the sample with 1% powder and 1.5% fat content. On day 7, the highest syneresis value was obtained for a plain sample with 2% fat content, and the lowest amount was computed for the plain sample with 1.5% fat content.

**TABLE 2:** Effects of various levels of beetroot extract microencapsulated powder addition on the syneresis value of yogurts with different fat content

Treatment		Storage time (day)			
Fat (%)	Betalains (%)	Day 1	Day 3	Day 5	Day 7
1.5	0	10.13 ± 0.06^Af^	9.32 ± 0.16^Bf^	8.61 ± 0.01^Cg^	8 ± 0.01^Dh^
2	0	13.23 ± 0.23^Ca^	13.43 ± 0.31^Aa^	12.53 ± 0.06^Ab^	12.33 ± 0.15^Ba^
3.2	0	11.54 ± 0.01^Bd^	8.94 ± 0.03^Dg^	12.86 ± 0.17^Aa^	11.17 ± 0.15^Cc^
1.5	0.5	9.82 ± 0.28^Ag^	9.46 ± 0.40^Ae^	9.76 ± 0.22^Af^	9.88 ± 0.14^Ad^
1.5	1	7.87 ± 0.06^Bi^	7 ± 0.00^Dh^	7.47 ± 0.02^Ci^	8.27 ± 0.25^Ag^
2	0.5	12.10 ± 0.10^Ab^	11.41 ± 0.08^Bc^	10.83 ± 0.06^Ce^	9.33 ± 0.31^Df^
2	1	11.92 ± 0.01^Bc^	11.92 ± 0.07^Bb^	12.15 ± 0.01^Ac^	11.73 ± 0.06^Cb^
3.2	0.5	11.37 ± 0.12^Ae^	10.74 ± 0.05^Bd^	7.57 ± 0.06^Ch^	7 ± 0.00^Di^
3.2	1	8.80 ± 0.26^Ch^	6.76 ± 0.25^Di^	11.06 ± 0.05^Ad^	9.74 ± 0.21^Be^

Note

Different lowercase letters within the columns and different uppercase letters within the rows indicate significant difference (*p* < .05).

### 3.13 Viscosity

According to Table 3, increasing fat content solely increased the viscosity in all storage days. It could be associated with the reduction of syneresis values. It was observed that the addition of powder, although increased the viscosity, was limited to a sample with 1.5% of fat content and 1% of the powder. In the rest of the samples, the effect of fat content was dominant. Fat globules make a matrix where water is entrapped. Possibly, the addition of powder maintained this matrix and reinforced the structure, and thereby, the syneresis was reduced, and subsequently, the viscosity was increased.

**TABLE 3:** Effects of various levels of beetroot extract microencapsulated powder addition on the viscosity of yogurt with different fat content

Treatment		Storage time (day)			
Fat (%)	Betalains (%)	Day 1	Day 3	Day 5	Day 7
1.5	0	2762 ± 2^Ah^	2552 ± 3^Bg^	2031 ± 2^Ci^	1911 ± 3^Di^
2	0	3350 ± 0^Ac^	2890 ± 2^Bc^	2583 ± 0^Bc^	2313 ± 3^Cd^
3.2	0	3807 ± 6^Ab^	3214 ± 4^Ba^	2921 ± 18^Ca^	2522 ± 3^Db^
1.5	0.5	2752 ± 3^Ai^	2333 ± 3^Bh^	2155 ± 5^Cg^	1919 ± 2^Dh^
1.5	1	2910 ± 10^Af^	2663 ± 3^Bf^	2133 ± 3^Ch^	1988 ± 3^Dg^
2	0.5	3852 ± 2^Aa^	3122 ± 3^Bb^	2916 ± 1^Cb^	2773 ± 2^Da^
2	1	2816 ± 6^Ag^	2329 ± 2^Bi^	2313 ± 12^Cf^	2117 ± 0^Df^
3.2	0.5	2970 ± 5^Ae^	2783 ± 2^Bd^	2467 ± 0^Cd^	2213 ± 2^De^
3.2	1	3008 ± 7^Ad^	2722 ± 3^Be^	2434 ± 2^Ce^	2318 ± 3^Dc^

Note

Different lowercase letters within the columns and different uppercase letters within the rows indicate significant difference (*p* < .05).

### 3.14 Color

According to the results presented in Table 4, addition of beetroot microencapsulated extract to yogurt reduced the L* values for samples incorporated with 0.5 and 1% of powder (at constant fat content). Based on our investigation, the lowest L* value was obtained for a sample with 2% fat content and 1% powder. Reduction in L* value was related to increased redness of produced powder and its effect on the final yogurt color. On the other hand, the addition of powder led to enhancement in a* values. A significant difference was observed between the plain sample and samples incorporated with 0.5 and 1% powder (*p* < .05). The highest a* value was attributed to the sample with 2% fat content and 1% powder. The lowest value was obtained for the plain sample. Indeed, an increase in a* value was attributed to the inherent red color of BEP and its produced microencapsulated powder. The inclusion of powder reduced the b* values of samples with constant fat content. The highest b* value was attributed to the plain sample, and the lowest was related to the sample with 1.5% fat content and 1% powder.

**TABLE 4:** Effect of beetroot extract microencapsulated powder addition on the color factors of yogurt with different fat content

Treatment		Color parameter		
Fat (%)	Betalains (%)	L*	a*	b*
1.5	0	89.75 ± 0.01^a^	−0.86 ± 0.01^g^	10.15 ± 0.00^a^
2	0	89.75 ± 0.01^a^	−0.86 ± 0.00^g^	10.15 ± 0.00^a^
3.2	0	89.75 ± 0.00^a^	−0.86 ± 0.00^g^	10.15 ± 0.00^a^
1.5	0.5	83.30 ± 0.35^b^	6.73 ± 0.03^f^	5.72 ± 0.03^f^
1.5	1	79.73 ± 0.02^c^	10.65 ± 0.05^c^	5.35 ± 0.02^g^
2	0.5	83.43 ± 0.03^b^	6.80 ± 0.00^e^	6.50 ± 0.02^c^
2	1	79.00 ± 0.00^d^	11.31 ± 0.08^a^	6.00 ± 0.00^e^
3.2	0.5	83.64 ± 0.56^b^	6.98 ± 0.03^d^	6.77 ± 0.03^b^
3.2	1	79.61 ± 0.10^c^	11.13 ± 0.02^b^	6.15 ± 0.02^d^

Note

Different small letters indicate significant difference within the columns (*p* < .05).

### 3.15 pH and acidity

Based on the obtained results presented in Table 5, the inclusion of microencapsulated powder significantly reduced the pH of yogurt (*p* < .05). On day 1, the highest pH value was obtained for the plain sample with 3.2% of fat content, and the lowest values were measured for samples with 0.5 of powder and 2% of fat content and 2% of fat content, and 1% of the powder. On day 3, the highest amount of pH value was obtained for the plain sample with 3.2% fat content, and the lowest amount was measured for the sample with 2% of fat content and 0.5% of the powder. On day 5, the lowest pH amount was related to the sample with 2% fat content and 0.5% powder, while the highest amount was related to the sample with 3.2% fat content and 0.5% powder. On day 7, the highest amount was obtained for the plain sample with 3.2% fat content, and the lowest amount was measured for the sample with 0.5 of powder and 2% fat content.

**TABLE 5:** Effect of beetroot extract microencapsulated powder addition on the acidity and pH values of yogurt with different fat content

Treatment		Acidity			
Fat (%)	Betalains (%)	Storage time (day)			
		Day 1	Day 3	Day 5	Day 7
1.5	0	0.68 ± 0.00^Db^	0.79 ± 0.03^Cab^	0.85 ± 0.00^Bb^	0.89 ± 0.01^Aa^
2	0	0.65 ± 0.00^De^	0.75 ± 0.06^Cbc^	0.87 ± 0.00^Ba^	0.88 ± 0.00^Aa^
3.2	0	0.56 ± 0.00^Dh^	0.65 ± 0.30^Ce^	0.78 ± 0.46^Bc^	0.81 ± 0.01^Ac^
1.5	0.5	0.68 ± 0.00^Cb^	0.80 ± 0.41^Aa^	0.76 ± 0.46^Bc^	0.80 ± 0.00^Ac^
1.5	1	0.65 ± 0.00^Dd^	0.73 ± 0.39^Cc^	0.27 ± 0.46^Bc^	0.83 ± 0.00^Ab^
2	0.5	0.67 ± 0.00^Bc^	0.75 ± 0.39^Ac^	0.26 ± 0.45^Ac^	0.79 ± 0.01^Ad^
2	1	0.73 ± 0.00^Ba^	0.75 ± 0.40^ABbc^	0.26 ± 0.45^ABc^	0.80 ± 0.00^Ac^
3.2	0.5	0.58 ± 0.00^Cg^	0.62 ± 0.33^Be^	0.23 ± 0.41^Ad^	0.69 ± 0.00^Af^
3.2	1	0.61 ± 0.00^Df^	0.69 ± 0.01^Cd^	0.68 ± 0.00^Bd^	0.70 ± 0.00^Ae^
		pH			
1.5	0	4.56 ± 0.00^Bde^	4.75 ± 0.00^Ad^	4.52 ± 0.00^Ce^	4.40 ± 0.00^Db^
2	0	4.57 ± 0.00^Ad^	4.68 ± 0.00^Ae^	4.53 ± 0.00^Ae^	4.51 ± 0.00^Aa^
3.2	0	4.95 ± 0.00^Ba^	5.12 ± 0.00^Aa^	4.83 ± 0.00^Cb^	4.72 ± 0.00^Da^
1.5	0.5	4.68 ± 0.00^Ac^	4.64 ± 0.00^Af^	4.47 ± 0.00^Bf^	4.41 ± 0.00^Bb^
1.5	1	4.69 ± 0.00^Bc^	4.87 ± 0.00^Ac^	4.68 ± 0.00^Bc^	4.63 ± 0.00^Ca^
2	0.5	4.55 ± 0.00^Bf^	4.59 ± 0.00^Ag^	4.33 ± 0.00^Cg^	4.33 ± 0.00^Cb^
2	1	4.55 ± 0.00^Bef^	4.75 ± 0.00^Ad^	4.46 ± 0.00^Cf^	4.40 ± 0.00^Db^
3.2	0.5	4.84 ± 0.00^Bb^	4.92 ± 0.00^Ab^	4.88 ± 0.00^Aa^	4.68 ± 0.00^Ca^
3.2	1	4.70 ± 0.00^Bc^	4.86 ± 0.00^Ac^	4.61 ± 0.00^Cd^	4.59 ± 0.00^Da^

Note

Different lowercase letters within the columns and different uppercase letters within the rows indicate significant difference (*p* < .05).

According to Table 5, the inclusion of powder enhanced the acidity of yogurt, and there was a significant difference between samples (*p* < .05). On day 1, the lowest acidity was related to the plains sample with 3.2% fat content, and the highest acidity was attributed to the sample with 1% of powder and 2% of fat content. On day 3, the sample obtained the lowest acidity with 3.2% of fat content and 0.5% of powder, while the highest acidity was measured for the sample with 1.5% of fat content and 0.5% powder. On day 5, the highest acidity was obtained for the plain sample with 2% of fat content, and the lowest amount was determined for the sample with 3.2% of fat content and 0.5% of the powder. On day 7, the highest acidity was determined for the plain sample with 1.5% of fat content, and the lowest value was measured for the sample with 3.2% of fat content and 0.5% of the powder.

## 4 CONCLUSION

Encapsulation of BE as an attractive source of anthocyanins presented positive results. The highest TPC was obtained at 15% MD, 7.5% GA, and 10% WPI. Also, the same results were achieved for antioxidant power. The optimum point was forecasted based on the highest TPC, antioxidant power, flowability, porosity, and lowest bulk density, yellowness, and moisture absorption, and thus, 15.61 MD, 9.19% GA, and 14.37% WPI were considered as an optimized point. All selected carriers had impressive impacts on preserving the bioactive phenolic and anthocyanin compounds, while MD, due to presenting a high *T_g_* point, led to less adhesion of microencapsulated powders to the drying chamber and thus, deterioration impacts were reduced. Also, the inclusion of obtained powders into yogurt led to a reduction in syneresis value.

## ETHICAL APPROVAL

Not applicable.

## CONFLICT OF INTEREST

The authors declare no conflict of interest relevant to this article.

## CONSENT TO PARTICIPATE

The authors declare their Consent to Participate in this article.

## CONSENT TO PUBLISH

The authors declare their Consent to Publish this article.

## Supporting information

Supplementary Material

## Data Availability

Not applicable.

## References

[fsn32804-bib-0001] Akhavan Mahdavi, S. , Jafari, S. M. , Assadpoor, E. , & Dehnad, D. (2016). Microencapsulation optimization of natural anthocyanins with maltodextrin, gum Arabic, and gelatin. International Journal of Biological Macromolecules, 85, 379–385. 10.1016/j.ijbiomac.2016.01.011 26772915

[fsn32804-bib-0050] AOAC (2000). Official Methods of Analysis 17th Ed, The Association of Official Analytical Chemists. Methods 925.10, 65.17, 974.24, 992.16.

[fsn32804-bib-0003] Arabpoor, B. , Yousefi, S. H. , Weisany, W. , & Ghasemlou, M. (2021). Multifunctional coating composed of *Eryngium campestre* L. essential oil encapsulated in nano‐chitosan to prolong the shelf‐life of fresh cherry fruits. Food Hydrocolloids, 111, 106394. 10.1016/j.foodhyd.2020.106394

[fsn32804-bib-0004] Assadpour, E. , & Jafari, S. M. (2019). Advances in Spray‐drying encapsulation of food bioactive ingredients: From microcapsules to nanocapsules. Annual Review of Food Science and Technology, 10, 103–131. 10.1146/annurev-food-032818-121641 30649963

[fsn32804-bib-0007] Burin, V. M. , Rossa, P. N. , Ferreira‐Lima, N. E. , Hillmann, M. C. R. , & Boirdignon‐Luiz, M. T. (2011). Anthocyanins: Optimisation of extraction from Cabernet Sauvignon grapes, microencapsulation and stability in soft drink. International Journal of Food Science & Technology, 46, 186–193.

[fsn32804-bib-0008] Cardoso‐Ugarte, G. A. , Sosa‐Morales, M. E. , Ballard, T. , Liceaga, A. , & San Martín‐González, M. F. (2014). Microwave‐assisted extraction of betalains from red beet (*Beta vulgaris*). LWT ‐ Food Science and Technology, 59, 276–282. 10.1016/j.lwt.2014.05.025

[fsn32804-bib-0011] Daoub, R. M. A. , Elmubarak, A. H. , Misran, M. , Hassan, E. A. , & Osman, M. E. (2018). 730 Characterization and functional properties of some natural Acacia gums. Journal of the Saudi Society of Agricultural Sciences, 17, 241–249. 10.1016/j.jssas.2016.05.002

[fsn32804-bib-0013] Fazaeli, M. , Emam‐Djomeh, Z. , Kalbasi Ashtari, A. , & Omid, M. (2012). Effect of spray drying conditions and feed composition on the physical properties of black mulberry juice powder. Food and Bioproducts Processing, 90, 667–675. 10.1016/j.fbp.2012.04.006

[fsn32804-bib-0014] Fazaeli, M. , Emam‐Djomeh, Z. , & Yarmand, M. S. (2016). Influence of Black mulberry juice addition and spray drying conditions on some physical properties of ice cream powder. International Journal of Food Engineering, 12, 277–285. 10.1515/ijfe-2015-0253

[fsn32804-bib-0017] Gengatharan, A. , Dykes, G. A. , & Choo, W. S. (2015). Betalains: Natural plant pigments with potential application in functional foods. LWT ‐ Food Science and Technology, 64, 645–649. 10.1016/j.lwt.2015.06.052

[fsn32804-bib-0018] Goula, A. M. , & Adamopoulos, K. G. (2005). Spray drying of tomato pulp in dehumidified air: II. The effect on powder properties. Journal of Food Engineering, 66, 35–42. 10.1016/j.jfoodeng.2004.02.031

[fsn32804-bib-0019] Goula, A. M. , & Adamopoulos, K. G. (2012). A new technique for spray‐dried encapsulation of lycopene. Drying Technology, 30, 641–652. 10.1080/07373937.2012.655871

[fsn32804-bib-0020] Jia, Z. , Dumont, M. J. , & Orsat, V. (2016). Encapsulation of phenolic compounds present in plants using protein matrices. Food Bioscience, 15, 87–104. 10.1016/j.fbio.2016.05.007

[fsn32804-bib-0021] Karaaslan, İ. , & Dalgıç, A. C. (2014). Spray drying of liquorice (*Glycyrrhiza glabra*) extract. Journal of Food Science and Technology, 51, 3014–3025. 10.1007/s13197-012-0847-0 26396294 PMC4571243

[fsn32804-bib-0023] Mahmoudi, P. , Khoshkhoo, Z. , Akhondzadeh Basti, A. , Mahasti Shotorbani, P. , & Khanjari, A. (2021). Effect of *Bunium persicum* essential oil, NaCl, Bile Salts, and their combinations on the viability of Lactobacillus acidophilus in probiotic yogurt. Quality Assurance and Safety of Crops & Foods, 13(1), 37–48. 10.15586/qas.v13i1.858

[fsn32804-bib-0024] Mansour, M. , Salah, M. , & Xu, X. (2020). Effect of microencapsulation using soy protein isolate and gum arabic as wall material on red raspberry anthocyanin stability, characterization, and simulated gastrointestinal conditions. Ultrasonics‐Sonochemistry, 63, 104927. 10.1016/j.ultsonch.2019.104927 31952001

[fsn32804-bib-0026] Mestry, A. P. , Mujumdar, A. S. , & Thorat, B. N. (2011). Optimization of spray drying of an innovative functional food: Fermented mixed juice of carrot and watermelon. Drying Technology, 29, 1121–1131. 10.1080/07373937.2011.566968

[fsn32804-bib-0028] Otálora, M. C. , Carriazo, J. G. , Iturriaga, L. , Nazareno, M. A. , & Osorio, C. (2015). Microencapsulation of betalains obtained from cactus fruit (*Opuntia ficus*‐*indica*) by spray drying using cactus cladode mucilage and maltodextrin as encapsulating agents. Food Chemistry, 187, 174–181. 10.1016/j.foodchem.2015.04.090 25977013

[fsn32804-bib-0029] Premi, M. , & Sharma, H. K. (2017). Effect of different combinations of maltodextrin, gum arabic and whey protein concentrate on the encapsulation behavior and oxidative stability of spray‐dried drumstick (*Moringa oleifera*) oil. International Journal of Biological Macromolecules, 105, 1232–1240. 10.1016/j.ijbiomac.2017.07.160 28757420

[fsn32804-bib-0032] Rezvankhah, A. , Emam‐Djomeh, Z. , & Askari, G. (2020). Encapsulation and delivery of bioactive compounds using spray and freeze‐drying techniques: A review. Drying Technology, 38, 235–258. 10.1080/07373937.2019.1653906

[fsn32804-bib-0033] Santana, A. A. , Cano‐Higuita, D. M. , De Oliveira, R. A. , & Telis, V. R. N. (2016). Influence of different combinations of wall materials on the microencapsulation of jussara pulp (*Euterpe edulis*) by spray drying. Food Chemistry, 212, 1–9. 10.1016/j.foodchem.2016.05.148 27374499

[fsn32804-bib-0041] Tiwari, B. K. , Patras, A. , Brunton, N. , Cullen, P. J. , & O’Donnell, C. P. (2010). Effect of ultrasound processing on anthocyanins and color of red grape juice. Ultrasonics Sonochemistry, 17(3), 598–604. 10.1016/j.ultsonch.2009.10.009 20015673

[fsn32804-bib-0035] Tolun, A. , Altintas, Z. , & Artik, N. (2016). Microencapsulation of grape polyphenols using maltodextrin and gum arabic as two alternative coating materials: Development and characterization. Journal of Biotechnology, 239, 23–33. 10.1016/j.jbiotec.2016.10.001 27720817

[fsn32804-bib-0038] Yousefi, S. , Emam‐Djomeh, Z. , Mousavi, S. M. A. , & Askari, G. R. (2012). Comparing the effects of microwave and conventional heating methods on the evaporation rate and quality attributes of pomegranate (*Punica granatum* L.) juice concentrate. Food and Bioprocess Technology, 5, 1328–1339. 10.1007/s11947-011-0603-x

[fsn32804-bib-0039] Zareifard, M. R. , Niakousari, M. , Shokrollahi, Z. , & Javadian, S. (2012). A feasibility study on the drying of lime juice: The relationship between the key operating parameters of a small laboratory spray dryer and product quality. Food and Bioprocess Technology, 5, 1896–1906. 10.1007/s11947-011-0689-1

[fsn32804-bib-0040] Zhu, F. (2017). Encapsulation and delivery of food ingredients using starch‐based systems. Food Chemistry, 229, 542–552. 10.1016/j.foodchem.2017.02.101 28372213

