# Supplementary material for: Yogurt fortification by microencapsulation of beetroot extract (Beta vulgaris L.) using maltodextrin, gum arabic, and whey protein isolate
Source: Food Sci Nutr. 2022 May 11;10(6):1875–87. doi: 10.1002/fsn3.2804 (PMC9179156; doi:10.1002/fsn3.2804)
Supplement: Supplementary file 1 — Supplementary Material [file FSN3-10-1875-s001.doc]

**Yogurt fortification by microencapsulation of beetroot extract (*Beta vulgaris* L.) using maltodextrin, gum Arabic, and whey protein isolate**

Shima Yousefi^a^* , Mohammad Kavyanirad^a^ , Mehrnaz Aminifar^b^, Weria Weisany^a^, Amin Mousavi Khaneghah^c^

^a^ Department of Agriculture and Food Science, Islamic Azad University, Science and Research Branch, Tehran, Iran.

^b^ Standard Research Institute – SRI, Karaj, Iran

^c^ Department of Food Science and Nutrition, Faculty of Food Engineering, University of Campinas (UNICAMP), Campinas, São Paulo, Brazil

*Corresponding author:

Shima Yousefi: shyousefi81@gmail.com

Short title: Yogurt fortification by microencapsulation of beetroot extract

Supplementary

Extraction of anthocyanins

According to the procedure reported in the literature, the conventional maceration method was applied to extract anthocyanins (Tiwari andand Cullen, 2012). For this aim, the grounded red beetroot was mixed with 30% v/v ethanol solution in a closed container. Maceration was continued for 2 h under constant stirring at 50 °C. Then, the container was kept in the dark for 2 h for the most efficient extraction/diffusion of residual anthocyanins in the plant matrix. In the next step, the obtained suspension was filtered with Whatman filter paper 1, and the extract was concentrated to 10° Brix by evaporator under a vacuum condition at 40 °C.

Determination of encapsulation efficiency (EE%)

Encapsulation efficiency was determined according to the method announced by Otálora et al. (2015). Total betalains content (TBC) and surface betalains content (SBC) were calculated for this test. To compute TBC, 100 mg of powders were weighed in the 2 mL tubes, and then 1 mL of distilled water was added. In the next step, samples were vigorously vortexed to have the walls of microcapsules broken down. Afterward, 10 mL of ethanol was added to samples to extract the entrapped and surface betalains (5 min), and then samples were filtered. To determine the SBC, microcapsules were rapidly washed (10 s) with 10 mL of ethanol in a vigorous form and then centrifuged at 3000 x g for 3 min. The obtained supernatant was collected and filtered through the Millipore membrane with 0.45 µm of particle size. The quantification of betalains was conducted using the spectrophotometry method at 536 nm. The obtained data were expressed using betanin as reference compound ($\varepsilon$ = 65,000 L/mol cm; molar mass = 550.1 g/mol, and $\lambda$ = 536 nm) (Coria Cayupán *et al.* 2011; Otálora *et al.* 2015). In more detail, quantification was carried out using the pH-differential method described by AOAC as explained by Akhavan Mahdavi et al. (2016). Two buffer systems were used: potassium chloride buffer at pH 1 (0.025 M) and sodium acetate buffer at pH 4.5 (0.4 M). An aliquot of the extract was transferred to a 10 mL volumetric flask and made up to 10 mL with the corresponding buffer, and the absorbance was read at 520 and 700 nm by a UV-vis spectrophotometer. TBC was calculated as betanin (betanidin 5-O-β- d -glucoside) according to the following equation:

$TBC \left( \frac{mg}{L} \right)=\Delta A\varepsilon\times1\times M\times103\times D$ (1)

where ∆A, (A520 pH 1-A700 pH 1) – (A520 pH 4.5-A700 pH 4.5); $\varepsilon$ (molar extinction coefficient = 65,000 L/mol cm for betanin; 1, path length in cm; M (molecular weight) = 550.1 g/mol for betanin; D, dilution factor; 103, conversion from gram to milligram.

Consequently, EE% was determined according to the following equation (Akhavan Mahdavi *et al.* 2016):

$EE\%=\frac{TBC-SBC}{TBC}\times100$ (2)

Bulk density

2 g of samples were weighed and poured in a 10 mL graduated cylinder and then kept for 1 min on a vibration vortex. The weight of powders to the volume occupied in the cylinder was bulk density (g/mL) (Karaaslan and Dalgıç, 2014).

Solubility

2 g of powders was added to 100 mL distilled water in a beaker and mixed using a homogenizer at 15000 rpm for 5 min. The obtained mixture was centrifuged at 3000g, and the separated supernatant (25 mL) was poured into a pre-weighed plate. Then, the plate was transferred to an oven set at 105 °C for 4 h for drying. The solubility was computed based on the difference between the obtained weight (Fazaeli *et al.* 2016).

Hygroscopicity

1 g of samples was weighed in a container with a certain weight and then placed in a desiccator containing a saturated solution of NaCl at ambient temperature. After a week (moisture equilibrium), the containers were weighed, and the moisture adsorption was determined by the following equation (Kurozawa *et al.* 2009):

$Hy (\%)=\frac{((W_{2}-W_{1}-W_{0})\times1000)+(W_{1}\times M)}{(W_{2}-W_{0})\times10}$ (4)

Hy (%) was moisture adsorption (g of adsorbed moisture/100 g powder); w_0_ was empty container (g); w_1_ was the weight of power (g); w_2_ was the weight of powder with container after moisture equilibrium; M was moisture content of powder (g/kg powder).

Flowability

The flowability of powders was calculated according to the Hausner ratio. 10 g of powders were weighed in a graduated cylinder. The initial volume was noted (Vb), and then the cylinder was tapped to reach a constant volume (Vf). Finally, the flowability was calculated by the equation below ( Freeman, 2007; Akhavan Mahdavi *et al.* 2016):

$Hausner ratio \left( HR \right)=(\frac{Vb}{Vf})\times100$ (5)

Flowability is denoted based on the different ranges of Hausner ratio:

HR between 1-1.1: free-flowing powder; HR between 1.1-1.25: medium flowing powder; HR between 1.25-1.4: difficult flowing powder; HR higher than 1.4: very difficult flowing powder.

Antioxidant power

The antioxidant power of powder samples was determined according to the method reported by Yousefi et al. (2012) with slight modification. 2, 2-Diphenyl-1-picrylhydrazyl (DPPH) was used as free reactive radicals to evaluate the antioxidant activity. 0.5 g of samples were dissolved in 25 mL of methanol, and time was given to extract anthocyanins. Then, the obtained suspensions were centrifuged, and the supernatants were separated. In the next step, 1 ml of supernatant was mixed with 3 mL of DPPH solution (0.025 ppm), and the reaction was conducted for 40 min in a dark place. Finally, the change in the absorption of solutions at 515 nm was recorded by using spectrophotometer UV-vis. Radical scavenging activity power was calculated by the equation below:

$DPPH \left( \% \right)=\frac{A_{s}}{A_{b}}\times100$ (6)

Where A_s_ and A_b_ were the absorbances of sample and blank solutions, respectively.

Total phenol content (TPC)

TPC of powder samples was measured by the Folin–Ciocalteu method based on that announced by Bansal et al. (2014) with brief modification. 0.5 g of powder was dissolved in 25 mL of methanol, and after a while, polyphenols were extracted; the solutions were centrifuged for 10 min. About 0.5 mL of supernatant was mixed with 2.5 ml of Folin Ciocalteu reagent 0.2 N, and the reaction was conducted for 5 min. Then, 2 mL of sodium carbonate solution (75 g/L) was added to the reaction mixture, and the volume was reached 25 mL by the addition of distilled water. The obtained solutions were placed at ambient temperature for 2 h, and the absorbance of samples was read using spectrophotometer UV-vis at 760 nm. Gallic acid solutions (0-100 mg/L) were used to plot the standard diagram. TPC was expressed as mg Gallic acid equilibrium/g of powder (GAE/g powder). All determinations were implemented in 3 replications.

**References**

Akhavan Mahdavi S, Jafari S M, Assadpoor E and Dehnad D (2016) Microencapsulation optimization of natural anthocyanins with maltodextrin, gum Arabic, and gelatin. *International Journal of Biological Macromolecules* 85 379–385.

Fazaeli M, Emam-Djomeh Z, and Yarmand M S (2016) Influence of Black mulberry juice addition and spray drying conditions on some physical properties of ice cream powder. *International Journal of Food Engineering* 12 277–285.

Karaaslan İ and Dalgıç AC (2014) Spray drying of liquorice (*Glycyrrhiza* *glabra*) extract. *Journal of Food Science and Technology* 51 3014–3025.

Kurozawa L E, Park K J and Hubinger M D (2009) Effect of carrier agents on the physicochemical properties of a spray-dried chicken meat protein hydrolysate. *Journal of Food Engineering* 94 326–333.

Tiwari B K and Cullen P J (2012) Extraction of red beet pigments. In *Red Beet Biotechnology: Food and Pharmaceutical Applications*.
